# Alanine Aminotransferase Elevation in Obese Infants and Children: A Marker of Early Onset Non Alcoholic Fatty Liver Disease

**DOI:** 10.5812/hepatmon.14112

**Published:** 2014-04-07

**Authors:** Guido Engelmann, Georg Friedrich Hoffmann, Juergen Grulich-Henn, Ulrike Teufel

**Affiliations:** 1 Department of Pediatrics, Lukas Hospital, Neuss, Germany; 2 Department of Pediatrics, University of Heidelberg, Heidelberg, Germany

**Keywords:** Liver, Obesity, Fatty Liver, Non-alcoholic Fatty Liver Disease, Child

## Abstract

**Background:**

Elevated aminotransferases serve as surrogate markers of non-alcoholic fatty liver disease, a feature commonly associated with the metabolic syndrome. Studies on the prevalence of fatty liver disease in obese children comprise small patient samples or focus on those patients with liver enzyme elevation.

**Objectives:**

We have prospectively analyzed liver enzymes in all overweight and obese children coming to our tertiary care centre.

**Patients and Methods:**

In a prospective study 224 healthy, overweight or obese children aged 1 - 12 years were examined. Body Mass Index-Standard Deviation Score, alanine aminotransferase, aspartate aminotransferase and gamma-glutamyl-transpeptidase were measured.

**Results:**

Elevated alanine aminotransferase was observed in 29% of children. 26 % of obese and 30 % of overweight children had liver enzyme elevations. Obese children had significantly higher alanine aminotransferase levels than overweight children (0.9 vs. 0.7 times the Upper Limit of Normal; P = 0.04).

**Conclusions:**

Elevation of liver enzymes appears in 29 % obese children in a tertiary care centre. Absolute alanine aminotransferase levels are significantly higher in obese than in overweight children. Even obese children with normal liver enzymes show signs of fatty liver disease as demonstrated by liver enzymes at the upper limit of normal.

## 1. Background

Obesity leads to accumulation of triacylglycerol in hepatocytes. This may cause either Non-alcoholic fatty liver disease (NAFLD) or Non-alcoholic steatohepatitis (NASH). There is strong evidence that in NAFLD patients insulin does not suppress lipolysis strong as it does in non-obese patients (1) leading to accelerated hepatic free fatty acid supply (2). This imbalance between uptake, synthesis, export and oxidation of free fatty acids leads to accumulation of fat in the cytoplasm of the liver cell (3). Fatty liver disease is often associated with the metabolic syndrome defined as hyperinsulinemia or a fasting blood glucose level > 100 mg/dL or a blood glucose level > 200 mg/dL 2h after an oral glucose tolerance test plus either obesity, dyslipidemia or hypertension (4, 5). Furthermore children with NAFLD carry a higher risk of developing cardiovascular diseases (6, 7) or even hepatocellular carcinoma (8). In the diagnostic approach of fatty liver disease the first findings often are increased aminotransferase levels (9) although the sensitivity of serum ALT is low. A recent study by Schwimmer et al. demonstrated, a 57 % sensitivity of ALT for the detection of NAFLD or NASH (10) and NASH and NAFLD also appear in patients with normal AST or ALT (11). However abnormal aminotransferases in daily practice serve as surrogate markers for fatty liver disease (12) in most clinical settings and a liver biopsy is usually performed only in obese patients with elevated liver enzymes, asking for NAFLD, NASH, autoimmunhepatitis or Wilsons disease (10).

Different upper limits of normal (ULN) for aminotransferases are defined according to the age of the child and according to the test kit used for measurement (13, 14). Therefore a comparison of absolute levels of liver enzymes of children above age groups is only possible if a ratio of liver enzyme and ULN is build. 

NAFLD is defined as an accumulation of fat in the cytoplasm of the hepatocyte in absence of significant alcohol consumption. Histology is characterized by a macrovesicular hepatic steatosis (15). NASH is a liver disease in absence of significant alcohol consumption with accumulation of fat into the hepatocyte and signs of hepatic fibroses and/or inflammation (16). NASH can progress to liver cirrhoses in up to 10 % of adult patients and also in children, whereas NAFLD is believed to represent a benign form of fatty liver accumulation usually not progressing to cirrhosis (17, 18).

However, the real proportion of patients with NAFLD remains obscure because not all patients show an elevation of liver enzymes. Therefore, other methods to identify fatty liver disease have been used such as MRI, ultrasound and more recently elastography (19-22). However measurement of liver enzymes is still the most often used method for screening of larger populations.

## 2. Objectives

We conducted a prospective study to investigate the appearence of elevated ALT, AST and GGT in overweight or obese children under the age of 12 years. Aim of the study was to compare liver enzyme elevations in a pediatric obese population in age groups with different upper limits of normal.

## 3. Patients and Methods

Children between 1 and 12 years of age coming to our outpatient clinic for medical assessment of obesity between January 1999 and December 2008 were included into the study when their Body-Mass-Index Standard Deviation Score (BMI-SDS) exceeded 90th percentile. BMI-SDS was used for statistical analyses since BMI-values are age-depending during childhood and adolescents (23). Patients with a BMI-SDS of 1.3 to 1,96 (90-97th Percentile) were considered overweight and patients with a BMI-SDS of >1,96 (>97th Percentile) were defined as obese according to considerations of the German Working Group on overweight and obesity (24). The German threshold values for boys and girls differ from the US-American (overweight: USA 85-95th, Germany 90-97th percentile; obese: USA > 95th, Germany > 97th percentile) (25). Anthropometric data (age, body weight, height) were measured and fasting blood samples for measurement of ALT, AST and GGT were taken. All patients underwent physical examination by an experienced pediatrician. In those patients with elevated liver enzymes, a complete laboratory work up (alkaline phosphatase, lactate dehydrogenase, bilirubin, total protein, immunoglobulin, transglutaminase antibodies) was performed and infectious (hepatitis B, C), metabolic (coeruloplasmin, alpha 1 antitrypsin) and autoimmune diseases (IgG, anti nuclear antibodies, liver-kidney microsome, smooth muscle antibody) were excluded. A medical history concerning hepatotoxic drugs, corticosteroids and liver injures including liver surgery was undertaken in all patients. Due to the young age of patients we did not ask for alcohol ingestion. Patients were excluded if the medical evaluation revealed additional diseases and/or if other diseases were already known. Exceptions were metabolic disorders included in the metabolic syndrome, such as hypertriglyceridemia, low HDL cholesterol and type 2 diabetes mellitus, impaired fasting glucose or impaired oral glucose tolerance. Body mass index, BMI-SDS (26) and SDS for height and weight were calculated (27). Liver enzymes were measured at 37 °C and calculated to 25°C until 31st of March 2003 due to the standard procedure in all German laboratories until March 2003. From April 1st 2003 liver enzymes and normal values were measured at 37°C. Data are expressed as measured value in [U/L] divided by the upper limit of normal (ULN) [U/L]. The ULN for age is defined as 2 standard deviations or more above the mean of liver enzymes measured in a normal population. For normal values used for analyses see Table 1. Nonparametric comparison was made by chi-square test. Linear regression was calculated using SPSS 12.0 Software and significance was calculated with the Anova Test model. Informed consent was obtained from patients and parents.

**Table 1.** Liver Enzyme Normal Values for Age According to the Literature ^a^

Enzyme	Temp	0-1 Year	1-3 Years	3-6 Years	6-12 Years
**AST, U/L**	25°C	40	24	18	23
	37°C	82	48	36	47
**ALT, U/L**	25°C	29	18	16	21
	37°C	54	33	29	39
**GGT, U/L**	25°C	13			
	37°C	34			

^a^ Before April 1st 2003 enzyme levels of aminotransferases and GGT were measured at 37°C and calculated to an activity at 25°C. This procedure was than changed to the international standard procedure. To compare data before and after that change and to overcome the problem of age depending normal values ratios of enzyme level and the upper limit of normal of the enzyme was build.

## 4. Results

### 4.1. Patient Data

Between January 1999 and December 2008 224 patients (99 boys and 125 girls) were included into the study. Mean age at presentation was 8 years and 9 months. For anthropometric data see Table 2. The median BMI-SDS for girls and boys was 2.5 and 2.6 respectively. Significantly less boys than girls were overweight (11 boys (9%) vs. 28 girls (22%); P = 0.03). The median height SDS was 1.1 (IR 0.2-1.7) in boys and 0.9 (IR 0.3-1.5) in girls indicating accelerated growth due to obesity.

**Table 2.** Anthropometric Data of the Study Patients Divided by Gender ^a,b, c^

	Male (n = 99)	Female (n = 125)	All Patients (n = 224)
**Age, mo**	103 ± 36	106 ± 32	105 ± 33
**Overweight/Obese**	11 (9) / 88 (91)	28 (22) / 97 (78)	
**Weight, kg**	54 ± 19	52 ± 18	53 ± 18
**Weight SDS**	2.6 ±0.7	2.4 ± 0.7	2.5 ± 0.7
**Height, cm**	138 ± 22	139 ± 18	139 ± 20
**Height SDS**	1.1 ± 1.0	0.9 ± 1.0	0.9 ± 1.0
**BMI, kg/m**^**2**^	26.9 ± 4.3	26.0 ± 4.1	26.4 ± 4.2
**BMI SDS**	2.6 ± 0.6	2.5 ± 0.6	2.5 ± 0.6

^a^ Abbreviations: BMI, body mass index; SDS, standard deviation score.

^b^ Data are presented as mean ± SD or No. (%).

^c^ The groups did not differ significantly according to weight, height and BMI.

### 4.2. Liver Enzymes

Elevated ALT was found in 66 patients (29%). The percentage of boys with elevated ALT was not significantly different to the percentage of girls with elevated ALT (30 % of boys, 29 % of girls). Of the 66 patients with elevated ALT 49 showed an isolated elevation of ALT. 9 patients showed a combination of ALT, AST and GGT elevation. In 7 patients only ALT and AST and in 1 patient ALT and GGT were elevated. None of the patients showed an isolated AST or GGT elevation: (Median AST: 0.6, IR 0:4-0:7 and GGT 0.6 IR: 0.4-0.7.)

**Figure 1.**
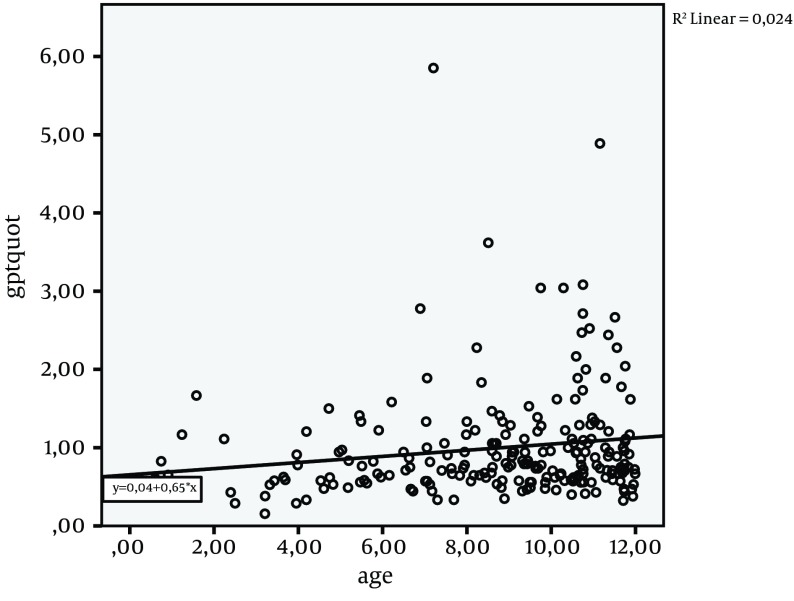
Alanine aminotransferase/Upper Limit of Normal-Ratio (ALT)/ULN-Ratio in Correlation With Age ALT/ratio levels above 1 are considered pathologic. The linear regression R square (Rsq) demonstrates no significant correlation of ALT/ULN and age.

**Figure 2.**
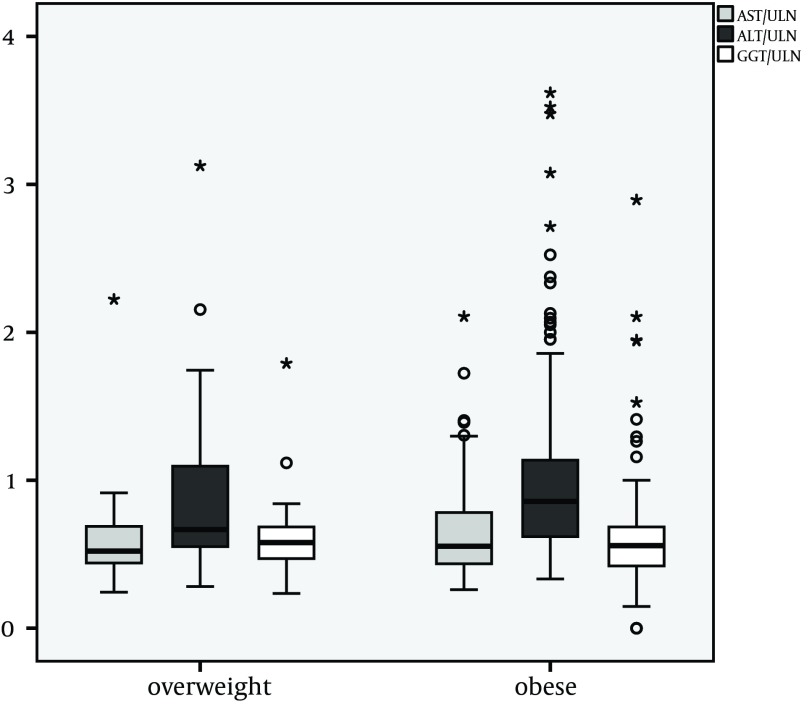
Median and Quartiles of Ratios of alanine aminotransferase (ALT), aspartate aminotransferase (AST) and gamma-glutamyl-transpeptidase (GGT) and the upper limit of normal (ULN) (n = 224) The difference between the median ALT in overweight and obese patients was significant. * (P = 0.04).

The median ration of ALT and ULN was 0.8 (IR: 0.6-1.1). Linear regression of age (Figure 1) and ALT/ULN-ratio showed that ALT was not correlated with age. Comparison of overweight and obese children showed significantly higher ALT/ULN ratios in the obese (overweight/obese Median 0.7/0.9 IR 0.5-1.1/0.6-1.1; P = 0.04) compared to overweight patients (Figure 2). AST and GGT did not differ significantly in absolute height, comparing overweight and obese children. The Median AST/ULN in overweight and obese patients was 0.5 in both (Inter Quartile Range (IR) 0.4-0.7 and 0.4-0.8). The Median GGT /ULN ratio was 0.6 in both (IR 0.5-0.7 and 0.4-0.7). 26% of overweight children and 30 % of obese children showed an isolated elevation of ALT above ULN (n.s.). Therefore absolute number of patients with an elevation of ALT did not differ significantly in contrast to median levels of ALT (see above).

## 5. Discussion

The present study shows a high percentage of ALT elevations in obese prepubertal children. AST levels were not significantly elevated. As ULN for liver enzymes in children are age dependent a direct comparison across certain age groups is not possible. Therefore a ratio of liver enzyme and age specific upper limit of normal was calculated.

The extent of ALT elevation was significantly higher in obese compared to overweight patients while the percentage of overweight and obese patients with ALT elevation above the ULN did not differ significantly. This possibly demonstrates that the degree of obesity contributes to the degree of liver cell damage in fatty liver disease. A study by Franzese et al. revealed a similar elevation of aminotransferases in a sample of 75 obese prepubertal children (25%) as in our study (28). However, normal values used for comparison are not given in that paper and a comparison between overweight and obese patients was not done. A study in obese Turkish school children demonstrated a significant elevation of ALT and AST (29). But these children and adolescents were older than those in our study. The ratio of aminotransferase and ULN has not been calculated and thus the different age groups could not be compared. 

Studies in obese and overweight adolescents showed a clear correlation of male gender and elevated ALT. This phenomenon was demonstrated in normal adolescent populations (National Health and Nutrition Examination Survey 1999-2004) (30, 31). Jamali et al. demonstrated a significant association between bmi and ALT in healthy male and female individuals (32). We also detected such a difference in our adolescent patients (data not shown) but in the prepubertal children there was no significant difference. We hypothesize that the difference develops with puberty and that it reflects the different social behavior in adolescent boys and girls with a higher prevalence of alcohol consumption in boys and young men in comparison to girls and young women. In a study by Strauss et al alcohol consumption was the only dietary factor that was related to ALT elevation (33).

The phenomenon of elevated liver ALT was irrespective of age. Surprisingly our data do not demonstrate a significant rise in the ALT with age. We believe that one reason for this is the relatively short duration of obesity in young children. The age range we overlooked in our study was only 12 years and many patients present to us after a relatively (in comparison to adult patients) short history of obesity.

Some authors state that AST serves as a surrogate marker for NAFLD (34). In our setting AST was of limited value because it was elevated in only 8 % of patients and all of them had an ALT elevation. Elevation of liver enzymes in obese patients has a correlation with NAFLD and therefore with other features of the metabolic syndrome (35). Thus the finding of a high proportion of elevated ALT in young obese children suggests a high degree of patients with a metabolic syndrome even in this very young age group. Our data show for the first time that with age adjusted ULN the degree and the amount of possible NAFLD in children can be assessed.

Our tertiary care hospital serves as a centre for patients with childhood obesity. Therefore our data may not be representative for the population of obese children. Childhood obesity may lead to NAFLD in every age group. Children are no exception from this rule. From our data it can be concluded, that fatty liver disease appears in very young children with obesity and that the degree of obesity contributes to the extent of fatty liver disease in this age group. We conclude that in every obese child measurement of ALT should be performed to identify those at risk for early NAFLD. Population based studies are needed to assess the real prevalence of children with fatty liver disease.
